# In vivo experimental study of anterior cervical fusion using bioactive polyetheretherketone in a canine model

**DOI:** 10.1371/journal.pone.0184495

**Published:** 2017-09-08

**Authors:** Takayoshi Shimizu, Shunsuke Fujibayashi, Seiji Yamaguchi, Bungo Otsuki, Yaichiro Okuzu, Tomiharu Matsushita, Tadashi Kokubo, Shuichi Matsuda

**Affiliations:** 1 Department of Orthopedic Surgery, Kyoto University Graduate School of Medicine, Kyoto, Japan; 2 Columbia University Medical Center, The Spine Hospital, New York-Presbyterian Healthcare System, New York, NY, United States of America; 3 Department of Biomedical Sciences, College of Life and Health Sciences, Chubu University, Aichi, Japan; University of Notre Dame, UNITED STATES

## Abstract

**Background:**

Polyetheretherketone (PEEK) is a widely accepted biomaterial, especially in the field of spinal surgery. However, PEEK is not able to directly integrate with bone tissue, due to its bioinertness. To overcome this drawback, various studies have described surface coating approaches aimed at increasing the bioactivity of PEEK surfaces. Among those, it has been shown that the recently developed sol-gel TiO_2_ coating could provide PEEK with the ability to bond with bone tissue *in vivo* without the use of a bone graft.

**Objective:**

This *in vivo* experimental study using a canine model determined the efficacy of bioactive TiO_2_-coated PEEK for anterior cervical fusion.

**Methods:**

Sol-gel–derived TiO_2_ coating, which involves sandblasting and acid treatment, was used to give PEEK bone-bonding ability. The cervical interbody spacer, which was designed to fit the disc space of a beagle, was fabricated using bioactive TiO_2_-coated PEEK. Both uncoated PEEK (control) and TiO_2_-coated PEEK spacers were implanted into the cervical intervertebral space of beagles (n = 5 for each type). After the 3-month survival period, interbody fusion success was evaluated based on μ-CT imaging, histology, and manual palpation analyses.

**Results:**

Manual palpation analyses indicated a 60% (3/5 cases) fusion (no gap between bone and implants) rate for the TiO_2_-coated PEEK group, indicating clear advantage over the 0% (0/5 cases) fusion rate for the uncoated PEEK group. The bony fusion rate of the TiO_2_-coated PEEK group was 40% according to μCT imaging; however, it was 0% of for the uncoated PEEK group. Additionally, the bone–implant contact ratio calculated using histomorphometry demonstrated a better contact ratio for the TiO_2_-coated PEEK group than for the uncoated PEEK group (mean, 32.6% vs 3.2%; p = 0.017).

**Conclusions:**

The TiO_2_-coated bioactive PEEK implant demonstrated better fusion rates and bone-bonding ability than did the uncoated PEEK implant in the canine anterior cervical fusion model. Bioactive PEEK, which has bone-bonding ability, could contribute to further improvements in clinical outcomes for spinal interbody fusion.

## Introduction

Anterior discectomy and fusion with autologous bone graft has been used for degenerative disc diseases in the cervical spine [1]. However, as the development of implant material research has progressed, surgeons have been able to choose from a variety of material options including allograft, titanium alloys, ceramics, and polymers. Polyetheretherketone (PEEK) implants have been widely used as intervertebral spacers since being approved as a medical-grade material by the Food and Drug Administration (FDA) in 1998 [2]. The elastic modulus of PEEK is close to that of human cortical bone. Despite this advantage in the aspect of the mechanical property, the downside is that PEEK is not able to integrate with bone tissue due to its bioinertness [3]. Recent clinical studies demonstrated that there is minimal evidence for better clinical and radiographic outcomes with the use of PEEK cages compared with bone grafts and titanium cages in the cervical spine [4].

There have been various types of *in vivo* experimental studies investigating the fusion rate of spinal implants [5]. Among these animal spine models, canine anterior cervical models are rarely reported and the fusion rates are shown to be suboptimal (0–75%) even with the use of an autologous bone graft, probably due to not only the wide range of motion of the canine cervical spine but also the primitive implant design without rigid initial fixation [6, 7]. The canine cervical spine is still an acceptable and tractable model for spinal applications; therefore, further research to improve the experimental model may contribute to a convincing *in vivo* evaluation of newly developed spinal implants.

In general, the use of neat PEEK in spinal fusion involves the simultaneous use of bone grafts inside the implants. However, problems related to the bone grafts (e.g., donor site issues and less storage of allografts, especially in Japan) compelled us to develop a graft-free interbody implant. Recently, a novel bioactive coating of sol-gel–derived TiO_2_ layer, which involves sandblasting and acid treatment, was developed to give PEEK bone-bonding ability in a simple and cost-effective way without affecting mechanical behavior [8]. This surface coating does not need high temperatures (exceeding glass transition temperature of PEEK) so the elastic modulus of PEEK cannot be altered [3, 9]. The sol-gel–derived TiO_2_ coating may possibly facilitate intervertebral fusion without any bone graft. The aim of this study was to determine the efficacy of bioactive TiO_2_-coated PEEK as a cervical intervertebral implant without a bone graft in a canine anterior cervical fusion model.

## Materials and methods

### PEEK implant fabrication

**Fig 1:**
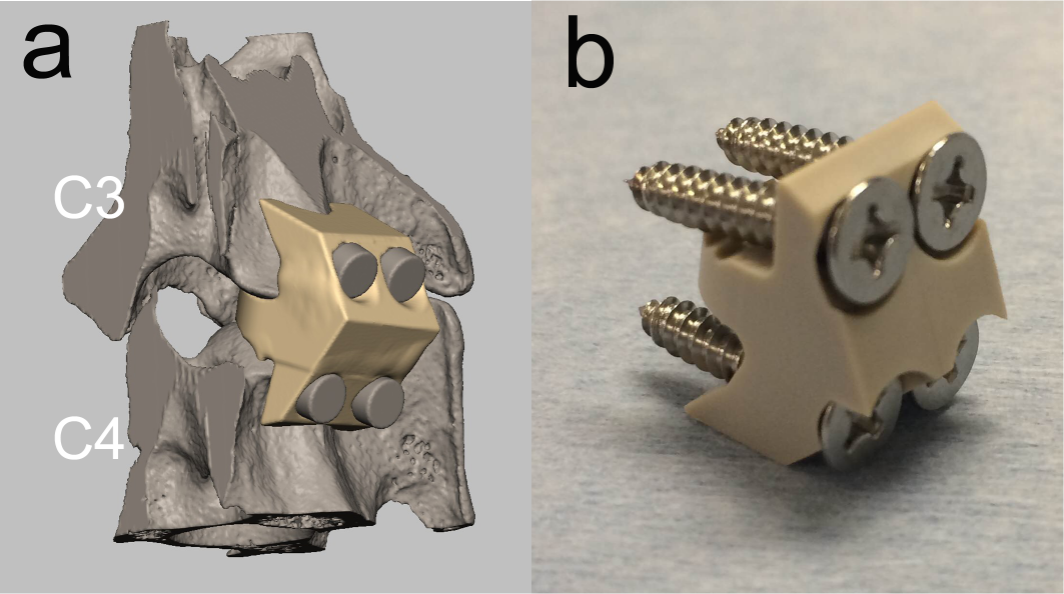
PEEK implant fabrication. (a) PEEK implant designed using computer-assisted design (CAD) software. (b) PEEK implant fabricated based on the CAD data.

A spacer-type interbody implant containing fixation screw holes was designed to fit the intervertebral space of the canine cervical spine (C3/4) by using three-dimensional (3-D) computer-aided design (CAD) software (Freeform, USA) (Fig 1A). Briefly, a rectangular clay construct with the same size as the vertebral body, was created using the software. Then, this construct was placed into the C3/4 intervertebral space and the redundant region other than the exact disc space was removed. Four screw holes were incorporated into the construct to enable rigid initial fixation. This construct designed for C3/4 was also applied to the C5/6 intervertebral space because it fit the space. Therefore, we used the same implant for both the C3/4 and C4/5 intervertebral spaces. Based on these CAD data, the PEEK spacer was fabricated by cutting a piece of PEEK block (Poisson’s ratio, 0.4; specific gravity, 1.3; flexural modulus, 4.2 GPa; tensile strength, 97 MPa) (TECAPEEK natural; Ensinger Gmbh, Nufringen, Germany) (Fig 1B). Four stainless screws with a diameter of 3.0 mm were used to fix the spacer to adjacent vertebrae.

### TiO_2_ coating on PEEK implant

Previously described TiO_2_ coating processes were utilized to give PEEK bioactivity [8]. PEEK implants were treated with sandblasting before the sol-gel coating method was used. Briefly, TiO_2_ particles with a median diameter of 7.62 μm (TOHO Titanium, Kanagawa, Japan) were blasted using a blast gun with a pressure of 0.5 MPa for 30 s. After sandblasting, PEEK implants were dipped in the TiO_2_ sol solution consisting of titanium tetraisopropoxide (TTIP), H_2_O, ethanol (EtOH), and nitric acid (HNO_3_) with a TTIP:H_2_O: EtOH:HNO_3_ molar ratio of 1:1:37:0.1. The implants were removed from the solution after 1 min at a rate of 1 cm/min and then air-dried at 80°C for 24 h. After drying, materials were soaked in 0.1 MHCl solution at 80°C for 24 h and then gently washed with ultrapure water.

The TiO_2_ particles that physically adhere to the PEEK surface chemically bond to the sol-gel–derived TiO_2_ layer. This chemical bonding provides sufficient stability of the sol-gel layer on PEEK [8].

### Surface characterization of PEEK implants

Surface morphology of the TiO_2_-coated PEEK and of the uncoated PEEK implants was examined by scanning electron microscopy (SEM) (S-4700; Hitachi Ltd, Tokyo, Japan). Additionally, the apatite (calcium phosphate) formation ability of the implant surface after soaking in the simulated body fluid (SBF), which predicts the *in vivo* bone-bonding ability (approved by ISO 23317)[10], was examined using SEM for one sample.

### Animals and surgical procedure

**Fig 2:**
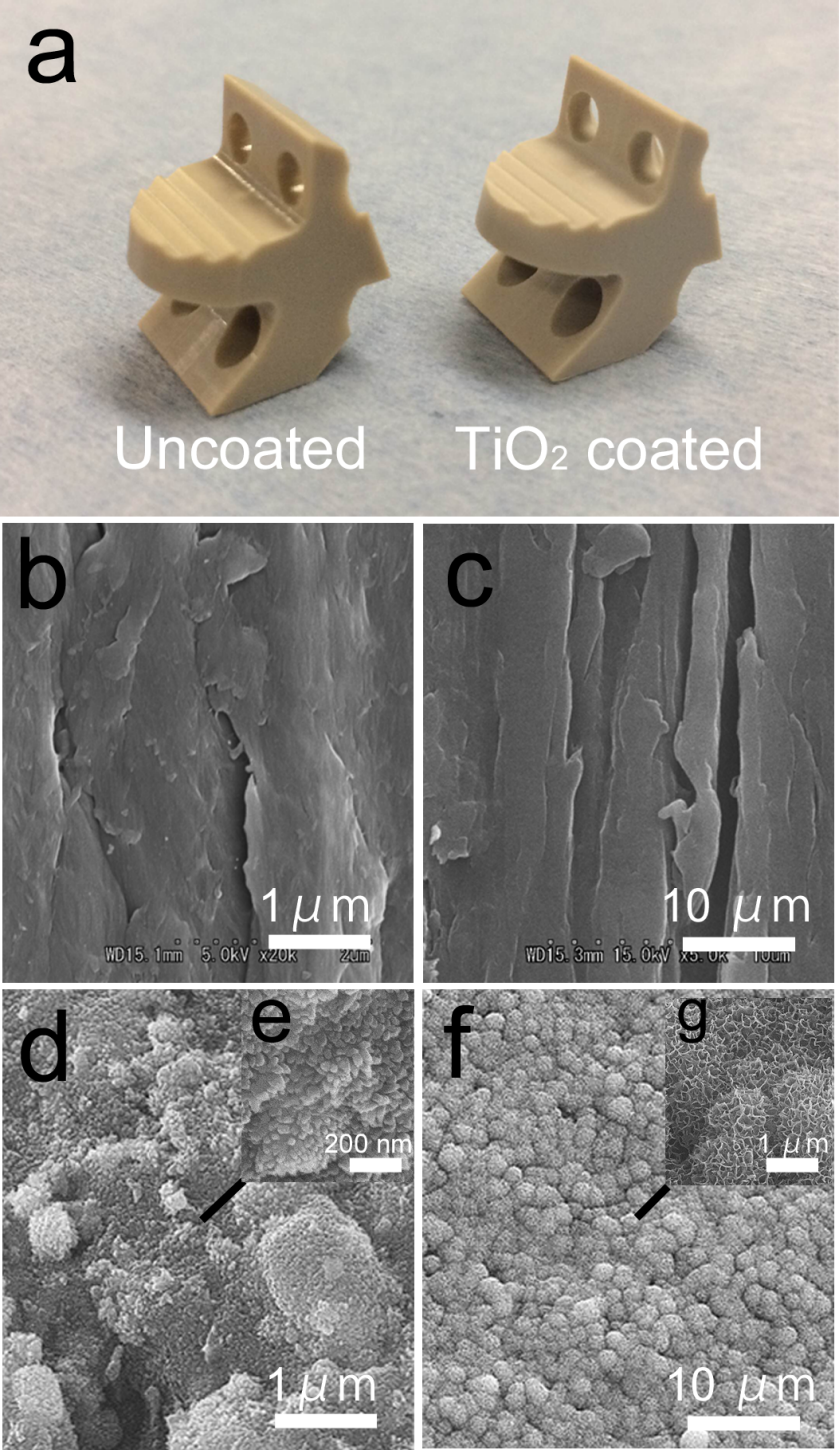
Macroscopic and SEM images of the PEEK implants. (a) Macroscopic view of the PEEK implants. The TiO_2_-coated PEEK is slightly white-tinged. (b) SEM image of the uncoated PEEK implant. (c) SEM image of the uncoated PEEK after soaking in the simulated body fluid (SBF). No apatite formation was observed on the surface. (d) SEM image of the TiO_2_-coated PEEK implant. (e) Magnified view. (f) SEM image of the TiO_2_-coated PEEK implant after soaking in the SBF. (g) Magnified view. Dome-shaped apatite formation was observed on the surface.

This animal study was approved by the Animal Research Committee of the Graduate School of Medicine, Kyoto University (Approval number #Medkyo 16173). Seven mature beagles (weight: 10–15 kg) were used. PEEK implants were sterilized with ethylene oxide gas before surgery. Surgery was performed under general anesthesia. Animals were sedated with intramuscular medetomidine and then intubated with a 7.5-mm tube and subsequently maintained with inhalation of isoflurane. After an approximately 10-cm midline incision was made above the top of the xiphoid process of the sternum, the sternohyoid muscle was divided at the midline. After protecting the trachea and esophagus, the longus colli was retracted and the anterior elements of the selected vertebral bodies were exposed. The discs were excised without removing the posterior longitudinal ligaments. Cartilagenous endplates were removed with a 1 mm high-speed steel burr with a diameter less than 1 mm. Meticulous attention was focused on the remaining bony endplates. After irrigation, either the TiO_2_-coated PEEK implant or the uncoated PEEK implant (control) was inserted into the intervertebral space (C3/4 or C5/6) with gentle impaction (animal assignments are shown in Table 1). All implants were able to fit the intervertebral space with slight extension of the neck. In three dogs, both implants were implanted in two levels to ethically decrease the animal number (Fig 2). After that, four stainless screws were inserted into adjacent vertebral bodies (two screws on each of the upper and lower vertebra) after gentle drilling. These screws were placed to reach the ventral cortex of the vertebra. Animals were administered intramuscular antibiotics for 3 days after surgery and kept in 1.0×1.5m cages with standard care. There were no restrictions postoperatively. At 3 months after surgery, animals were euthanized with intravenous pentobarbital sodium administration. Then, the specimens containing the fusion segment (the implant and the adjacent vertebrae) were harvested.

**Table 1:** Description of specimens and summary of biomechanical, radiographic, and histological analyses.

Specimen	Coating	Animal	Level	CT fusion	MP fusion	Histology BIC (%)	Immediate postoperative local lordosis (°)	Postoperative local lordosis at 3 months (°)
1	Uncoated	Dog 1	C3/4	-	-	0	8.0	5.5
2	Uncoated	Dog 4	C5/6	-	-	0	2.4	4.3
3	Uncoated	Dog 5	C5/6	-	-	8	3.8	2.5
4	Uncoated	Dog 6	C3/4	-	-	8	8.2	7.1
5	Uncoated	Dog 7	C5/6	-	-	0	2.1	3.1
6	TiO_2_-coated	Dog 1	C5/6	-	-	0	15.1	15.7
7	TiO_2_-coated	Dog 2	C3/4	+	+	42	1.6	2.2
8	TiO_2_-coated	Dog 3	C3/4	-	-	23	1.7	0.2
9	TiO_2_-coated	Dog 6	C5/6	-	+	56	20.6	17.9
10	TiO_2_-coated	Dog 7	C3/4	+	+	43	3.4	5.1

CT: computed tomography; MP: manual palpation; BIC: bone implant contact.

#### Radiographic analyses

X-ray examination was performed immediately after surgery and 3 months after surgery. During each time period, the local lordosis angle was measured (cobb angle between the superior endplate and inferior endplate of the fusion segment). A μ-CT scan (SMX-100CT-SV-3; Shimadzu Corp., Kyoto, Japan) with a slice thickness of 0.04 mm was performed after harvesting the specimen at 3 months after surgery. The presence of bony fusion was determined if the bony bridge formation existed between the superior vertebra and the inferior vertebra in the sagittal or coronal view of multi-planar reconstructed (MPR) μ-CT imaging (VG studio MAX 2.2; Volume Graphics GmbH, Heidelberg, Germany).

#### Manual palpation testing

After removing the soft tissues, each specimen was cut sagittally at the midline of the vertebral body with a diamond band-saw (BS-3000CP; EXACT cutting system, Norderstedt, Germany). Each bisected part was examined to determine whether the bone–implant gap was seen by manually providing extension force under stereomicroscopic observation. The extension force was applied by pinching both the upper and lower vertebra. If the bone–implant gap (approximately more than 100 μm) was observed at either interface (upper or lower vertebra), it was defined as “not fused.”

#### Histology and histomorphometry

After radiographic analyses and manual palpation, the specimens were fixed in 10% phosphate-buffered formalin (pH 7.25) for 7 days, dehydrated in serial concentrations of ethanol (70, 80, 90, 99, 100, and 100% vol/vol) for 3 days at each concentration, and embedded in polyester resin. Thick sections (250 μm) were cut with a band saw perpendicular to the axis of the implant. Seven sections were produced from each vertebra–implant specimen and sections 1 (left side), 4 (middle), and 7 (right side) were ground to a thickness of 80–100 μm using a slide grinding machine (Microgrinding MG-4000; EXACT). Then, they were stained with Stevenel blue and van Gieson picrofuchsin (orange-red, mineralized bone; blue, fibrous tissue; dark blue, cartilaginous tissue). Thorough microscopic analysis was performed for the histological slides using a transmitted light microscope (Eclipse 80i; Nikon, Tokyo, Japan) with a digital camera (DS-55M-L1; Nikon, Tokyo, Japan). The stained sections were evaluated by quantitative histomorphometry for the amount of direct bone contact with the PEEK implant surface and the bone–implant contact (BIC) ratio was determined using the two-dimensional (2-D) image processing software (Image J; National Institutes of Health, Bethesda, MA, USA). The BIC ratio was calculated after the tissue implant contact area had been manually defined by a blinded observer. Each histomorphometric value was expressed as the mean percentage value of the three sections.

#### Definition of fusion

In this study, fusion was defined if the specimen satisfied both of the following: (1) no bone–implant gap during manual palpation analysis and (2) at least one site of bony bridging on MPR μ-CT imaging or direct bone–implant contact in at least one section during histology.

### Statistical analysis

The Mann-Whitney U test and the χ^2^ test were used for continuous and categorical variables, respectively. JMP version 11 (SAS, Cary, NC) was used for all analyses and the significance was set at p < 0.05.

## Results

### Surface characterization of PEEK implants

**Fig 3:**
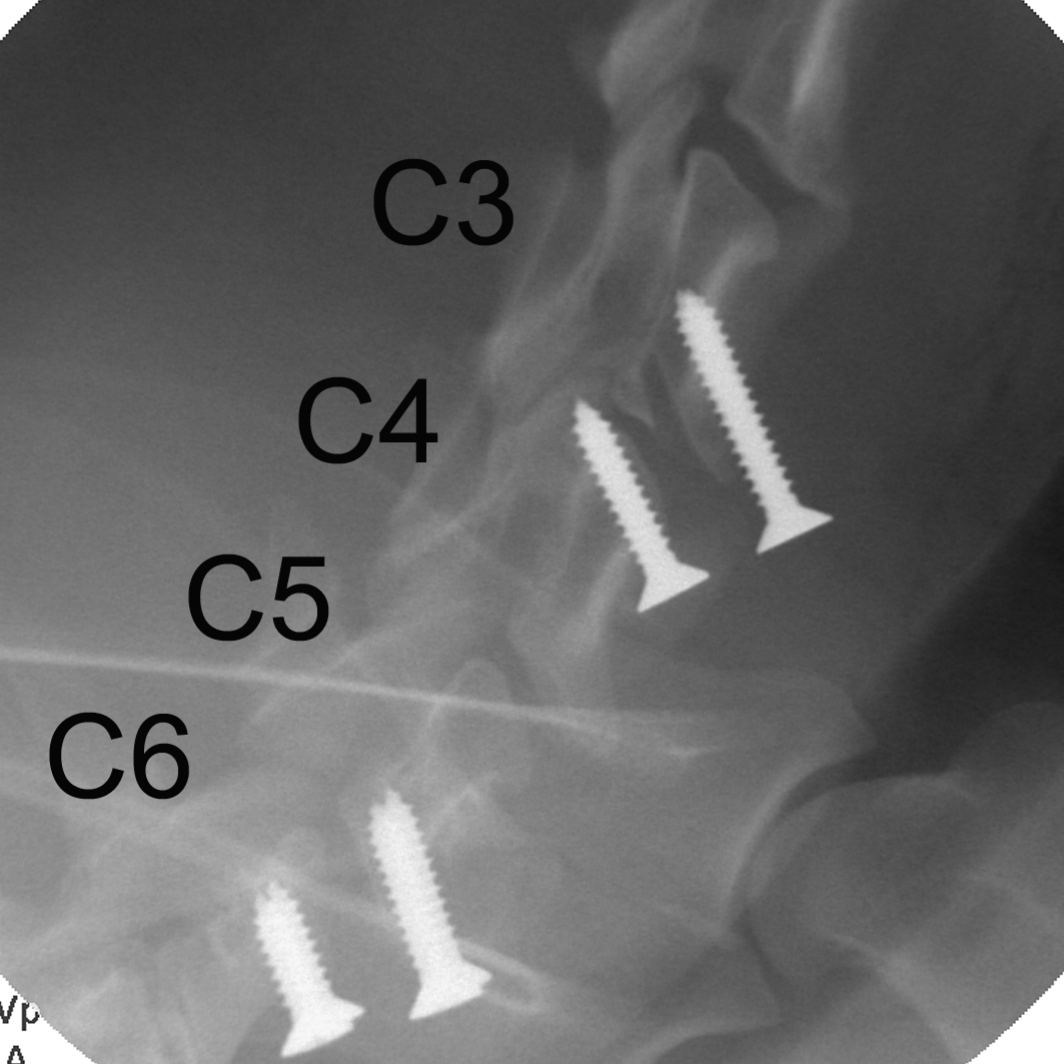
Postsurgical X-ray examination. PEEK spacers (radiolucent) were implanted and fixed with screws in the C3/4 and C5/6 intervertebral spaces.

The TiO_2_-coated surface was fully covered with an extremely thin white-tinged layer in the macroscopic view (Fig 3A). The SEM images of the surfaces of the PEEK implants are shown in Fig 3B–3G. The uncoated PEEK showed polishing traces on its surface (Fig 3B and 3C), whereas a uniform nanoscale sol-gel–derived TiO_2_ layer on the microscale-rough surface induced by sandblasting was observed on TiO_2_-coated PEEK (Fig 3D and 3E). Magnified images on any arbitrary points of the TiO_2_-coated PEEK implant showed the same findings, indicating that a uniform TiO_2_ coating was applied to the surface with sandblasting. In addition, dome-shaped apatite formation was observed after soaking in the SBF (Fig 3F and 3G), which predicts the *in vivo* bone-bonding ability. The uncoated PEEK implant did not demonstrate apatite layer formation after soaking in the SBF.

### In vivo experiment

A summary of implant assignments and the results of all analyses are shown in Table 1. All animals uneventfully underwent surgery except for one (dog 1) that experienced transient hind limb palsy but recovered by two days after surgery. No surgical site infection or dislodgement or migration of implants or screws had occurred at three months after surgery.

#### Radiographic analyses

**Fig 4:**
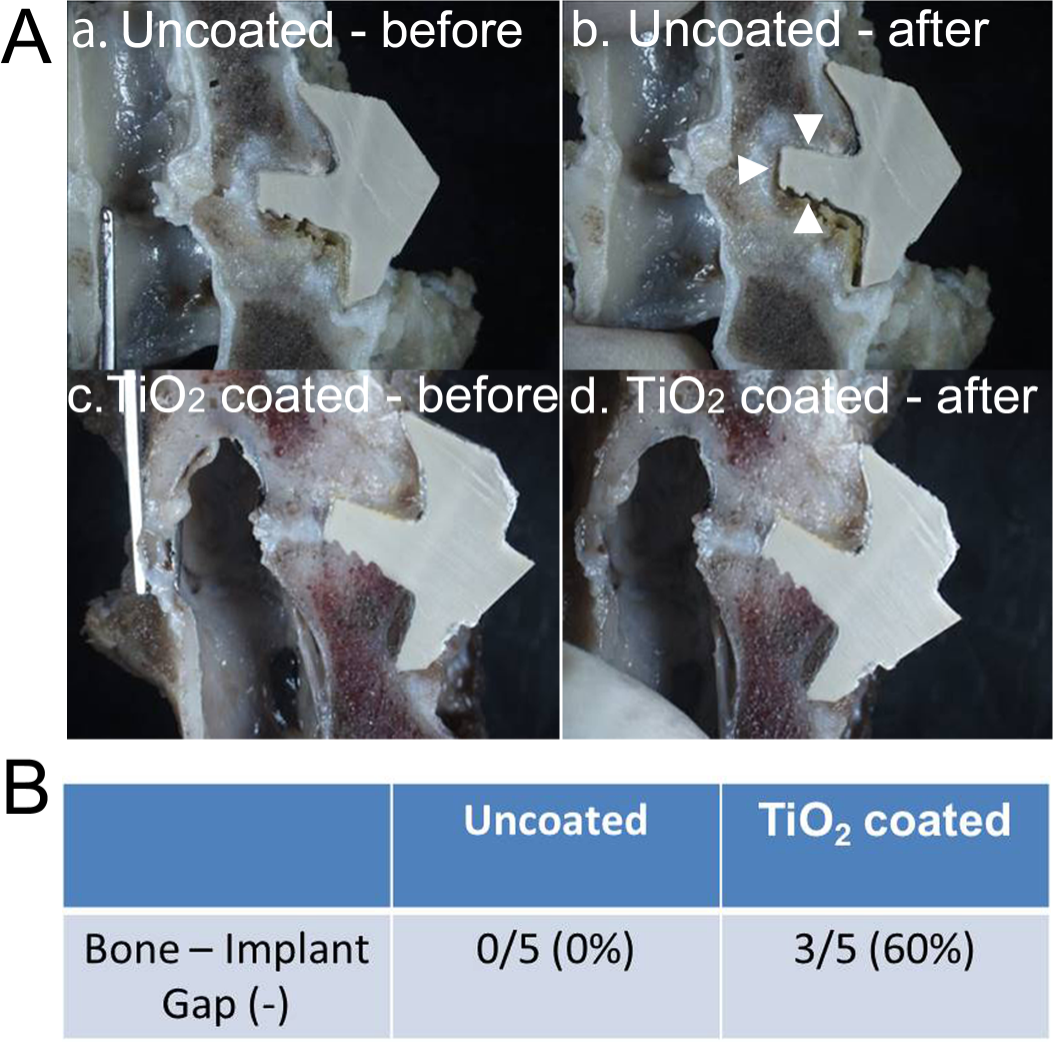
**(A) The** μ**-CT images.** (a) Uncoated PEEK. Bone cysts and osteolytic lesion were observed surrounding the PEEK implant (black arrowheads). (b) TiO_2_-coated PEEK. An intervertebral bony bridge was observed (white arrowheads). **(B) The bony union rate of the PEEK implants based on the** μ**-CT finding.**

The immediate postoperative and 3-month postoperative local lordosis angles were 6.6±6.4 (mean±SD) and 6.3±5.8, respectively, representing no significant angle loss during the postsurgical periods (p = 0.85) (Table 1). The μ-CT imaging showed a 40% (2/5 cases) bony fusion rate for the TiO_2_-coated group compared with a 0% bony fusion rate for the uncoated PEEK group (p = 0.113) (Fig 4A and 4B). Bone cysts and fibrous tissues were observed between implants and bone in three out of five specimens in the uncoated PEEK group. These vertebral endplate cysts might predict subsequent nonunion [11].

#### Manual palpation testing

**Fig 5:**
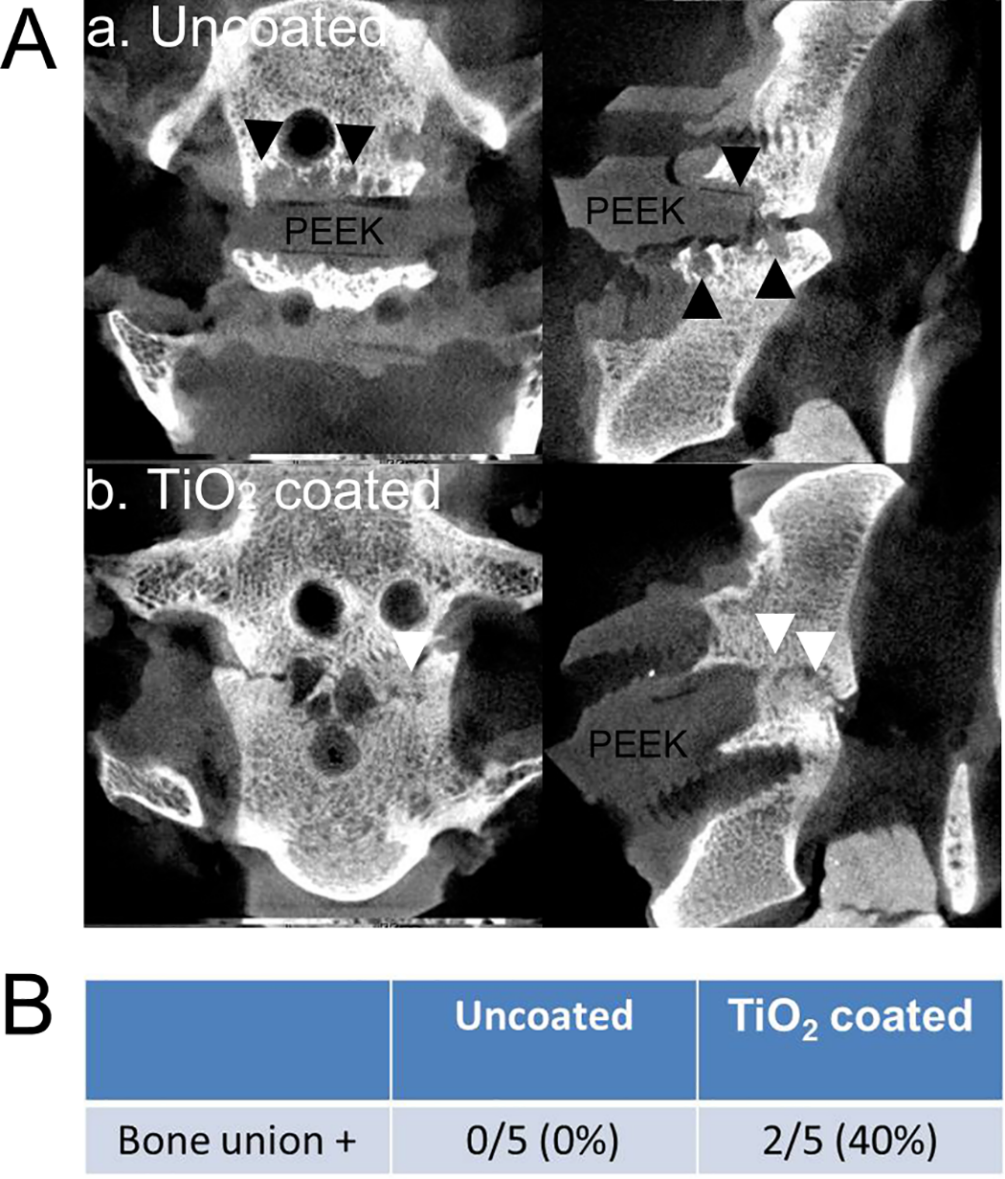
**(A) Manual palpation analysis**. (a, b) Uncoated PEEK. Bone–implant gap was observed after applying extension force (white arrowheads). (c, d) TiO_2_-coated PEEK. No bone–implant gap after extension force. (B) Fusion rate of the PEEK implants based on the manual palpation analysis.

Manual palpation analysis indicated a fusion (no gap between the bone and implants) rate of 60% (3/5 cases) for the TiO_2_-coated group, thereby indicating a clear advantage over the uncoated PEEK group, which had a 0% fusion rate (p = 0.038) (Fig 5A and 5B).

#### Histology and histomorphometry

**Fig 6:**
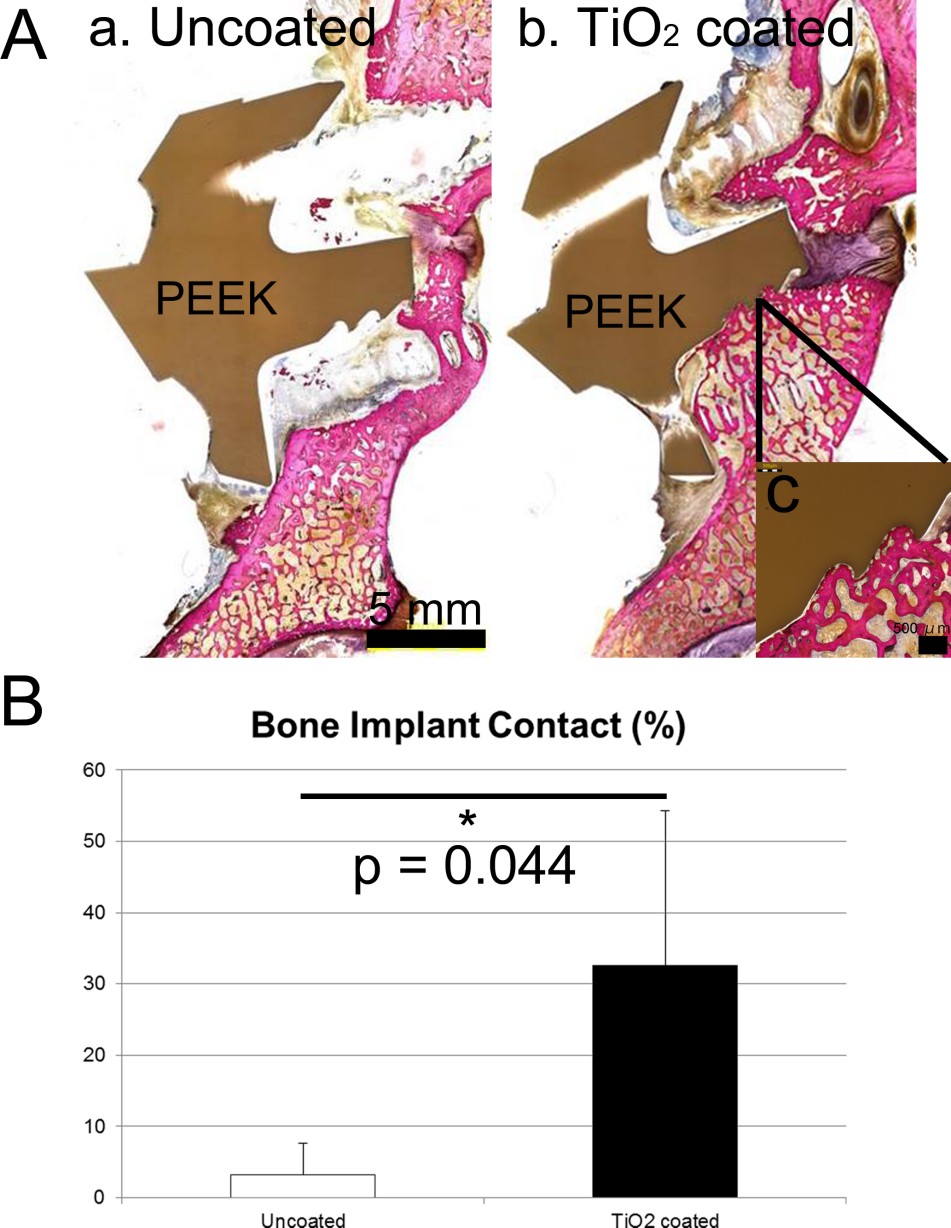
**(A) Histology.** (a) Uncoated PEEK. Osteolytic lesion was observed surrounding the PEEK implant. (b) TiO_2_-coated PEEK. Bone–implant integration was observed on the lower surface of the PEEK implant. Another section of this specimen showed direct apposition on the upper surface, but not on the lower surface. This specimen was considered to be fused because there was no gap during the manual palpation analysis. (c) Magnified view. (B) The bone–implant contact ratio based on histomorphometry.

Four of five specimens in the TiO_2_-coated PEEK group demonstrated direct bone–implant bonding, whereas direct bonding was rarely observed for the uncoated PEEK group. An osteolytic lesion was seen on the uncoated PEEK (Fig 6A). The BIC ratio (%) was significantly greater for the TiO_2_ PEEK group than for the uncoated PEEK group (32.6% vs 3.2%; p = 0.044) (Fig 6B).

## Discussion

This *in vivo* experimental study elaborated the efficacy of TiO_2_-coated PEEK with bone-bonding ability in a canine model. Additionally, newly designed spacer-type PEEK implants were used in the canine anterior cervical discectomy fusion (ACDF) model.ACDF is one of the most prevalent surgical procedures performed in the cervical spine especially for degenerative disc diseases. Since it was initially reported by Cloward, Smith and Robinson, ACDF with an autologous iliac bone graft has resulted in successful long-term clinical outcomes.[12] However, donor site morbidity and failure associated with graft collapse, subsidence, or resorption with subsequent pseudoarthrosis were also described in many reports.[13–16] In an effort to resolve these problems, Bagby developed a stainless steel cage with cancellous bone tips in 1988.[17] This cage named cage demonstrated good fusion rates for lumbar interbody fusion.[18] In subsequent years, titanium alloy cages were commercialized. Titanium cages in the cervical spine demonstrated high fusion rates and good surgical outcomes; however, some concerns still exist regarding high subsidence rates or stress shielding due to the high elastic modulus of titanium metal.[19, 20]

Since its commercial release in 1998, PEEK cages have become prevalent worldwide. PEEK has low elastic modulus compared with titanium alloys and can be reinforced to a value close to that of human bone by using carbon. Therefore, stress shielding could be avoided. Furthermore, radiolucency of PEEK allows less artifacts on CT and magnetic resonance imaging scans and allows easy visualization of the bony fusion status.[3, 21] However, due to the chemical inertness of the surface, PEEK does not integrate with bone tissue and often forms fibrous tissues on the bone–implant interface. Olivares-Navarrete et al reported that osteoblastic differentiation of progenitor cells was reduced on the PEEK surface and that inflammatory chemokines were produced, thereby contributing to fibrous tissue generation.[22] Theoretically, this bioinertness might contribute to consequent nonunion, which may limit successful outcomes. In fact, recent clinical studies reported that no differences in clinical outcomes were found between PEEK, titanium, and carbon fiber cages [4, 23]. There is still room for improvement in terms of implant surface modifications for better clinical outcomes.

Some potential solutions addressing the bioinertness of the PEEK surface have been reported. For instance, titanium or hydroxyapatite (HA) coating was reported to improve direct bone growth on the PEEK surface in an animal model [24, 25]. However, a relatively thick titanium layer with plasma spraying (ranging from 13.4 to 70 μm) creates concerns about delamination of the layer [26, 27], and extremely high temperatures during the coating process may result in denaturation of PEEK. HA coatings are susceptible to degradation over long-term implantation periods [28]. In contrast, a newly developed sol-gel–derived TiO_2_ coating utilized in the present study can provide an extremely thin (30 nm), durable, and uniform layer with a simple and cost-effective process [8]. In a previous study, we evaluated the bonding strength of the TiO_2_ gel layer to the PEEK with a modified ISO 2409 tape test [9]. A sol-gel–derived layer adhered to the substrate so firmly that the layer could not be peeled from the substrate by detaching the tape pressed to the surface due to its dramatically improved hydrophilicity. In addition, Patsi et al. suggested that the bonding strength of a sol-gel–derived layer was proven to be sufficient for their use as an implant coating material (>24 MPa) [29]. The chemical bonding between sandblasted TiO_2_ particles and the sol-gel layer allows strong bonding of the coating layer. Additionally, the temperature used in this method was significantly lower (80°C at maximum) than those used during traditional coating processes and did not adversely affect the mechanical property of PEEK. In terms of surface roughness, sandblasting and sol-gel treatment provide PEEK surface with nano-scale roughness (as observed in the SEM images). Khoury et al. utilized a neutral atom beam technique to produce nano-scale roughness on neat PEEK, which improved bone apposition in a rat calvarial model [30]. This type of nano-scale roughness can contribute to the fusion status in *in vivo* experiments.

The present *in vivo* study using a canine cervical spine model demonstrated better fusion rates and bone–implant contact ratios at 3 months after surgery, representing the efficacy of this TiO_2_ coating during the preclinical phase. The postsurgical period of 3 months was set in this pilot study to evaluate the early-phase fusion status. Based on this result, further study evaluating longer-term observations may be warranted. Canine models for spinal fusion are commonly used and are suitable for many different techniques and experimental variables.[5] However, the canine ACDF model has been reported in few studies. In one report, Shima et al investigated the availability of a synthetic tricalcium phosphate (TCP) in a mongrel ACDF model by using plain X-ray and histology.[6] The authors compared the fusion rate of a TCP dowel to an autograft and reported a 75% fusion rate even in the autograft group (0% in the TCP group). In another study, Cook et al evaluated the fusion status of a simple HA block with thorough analyses that provided biomechanical, radiographic, and histological findings for hounds.[7] They concluded that the HA spacer could be an alternative for autologous bone graft; however, some HA blocks cracked and replaced and clear quantitative fusion rates were not determined. Therefore, to date, there has been little knowledge about the fusion rate of the canine ACDF model. Given the greater range of motion of the canine cervical spine, it still remains challenging to acquire fusion without a bone graft in the canine ACDF model [5, 31]. We postulated that the fusion rate could be improved by using custom-made implants that provide rigid initial fixation. In the present study, the definition of fusion was clearly determined as “no bone–implant gap on manual palpation with bony bridging on CT or direct bone–implant contact in histology”. Takemoto et al. reported that manual palpation under stereomicroscopic observation enabled easy detection of non-fusion and (in this study as well) its results well-reflected the results observed during histological evaluation [32]. Under these strict criteria, the fusion rate was 60% for TiO_2_-coated PEEK even without bone graft, which is attributed to not only the strong bioactivity of the TiO_2_ coating but also the rigid initial fixation. There was no subsidence, loss of local lordotic angle or migration of implants. This may have resulted from the custom-made design of the implant to fit the intervertebral space, rigid fixation of screws incorporated into the implant, and perhaps PEEK’s ideal elastic modulus. A canine is considered easy to handle and its vertebrae are generally of a size that makes surgery simple. Therefore, the newly established implant design and canine ACDF model could play a valuable role in future experimental studies evaluating spinal applications.

This study had a few limitations. First, we implanted in two levels to ethically decrease the number of animals involved. This might have affected the fusion status due to the kinematic difference between the C3/4 and C5/6 levels. Second, normally, there was fibrous tissue in the interface wherein the gap was observed during manual palpation. Therefore, there was no influence on the analysis of the BIC ratio. However, extremely slight bone–implant contact (approximately less than 200 μm) that was not able to be observed under a stereomicroscope might have been delaminated during the manual palpation analysis. This indicates that we might have underestimated the BIC ratio.

## Conclusion

The custom-made PEEK spacer coated with a sol-gel–derived TiO_2_ layer enhanced bone–implant bonding and provide comparable rate of intervertebral fusion to the previous reports, even without bone grafts.

## Supporting information

S1 File Surface of TiO2-coated PEEK implant. (A) Macroscopic view. (B) SEM image (C) Magnified SEM images on each point (a, b, and c). Nano-scale roughness was observed on each point.
(TIF)

S2 File BIC ratio data. Supporting information file of Fig 6.
(XLSX)
