# Supplementary figures and images for: In vivo experimental study of anterior cervical fusion using bioactive polyetheretherketone in a canine model
Source: PLoS One. 2017 Sep 8;12(9):e0184495. doi: 10.1371/journal.pone.0184495 (PMC5590956; doi:10.1371/journal.pone.0184495)

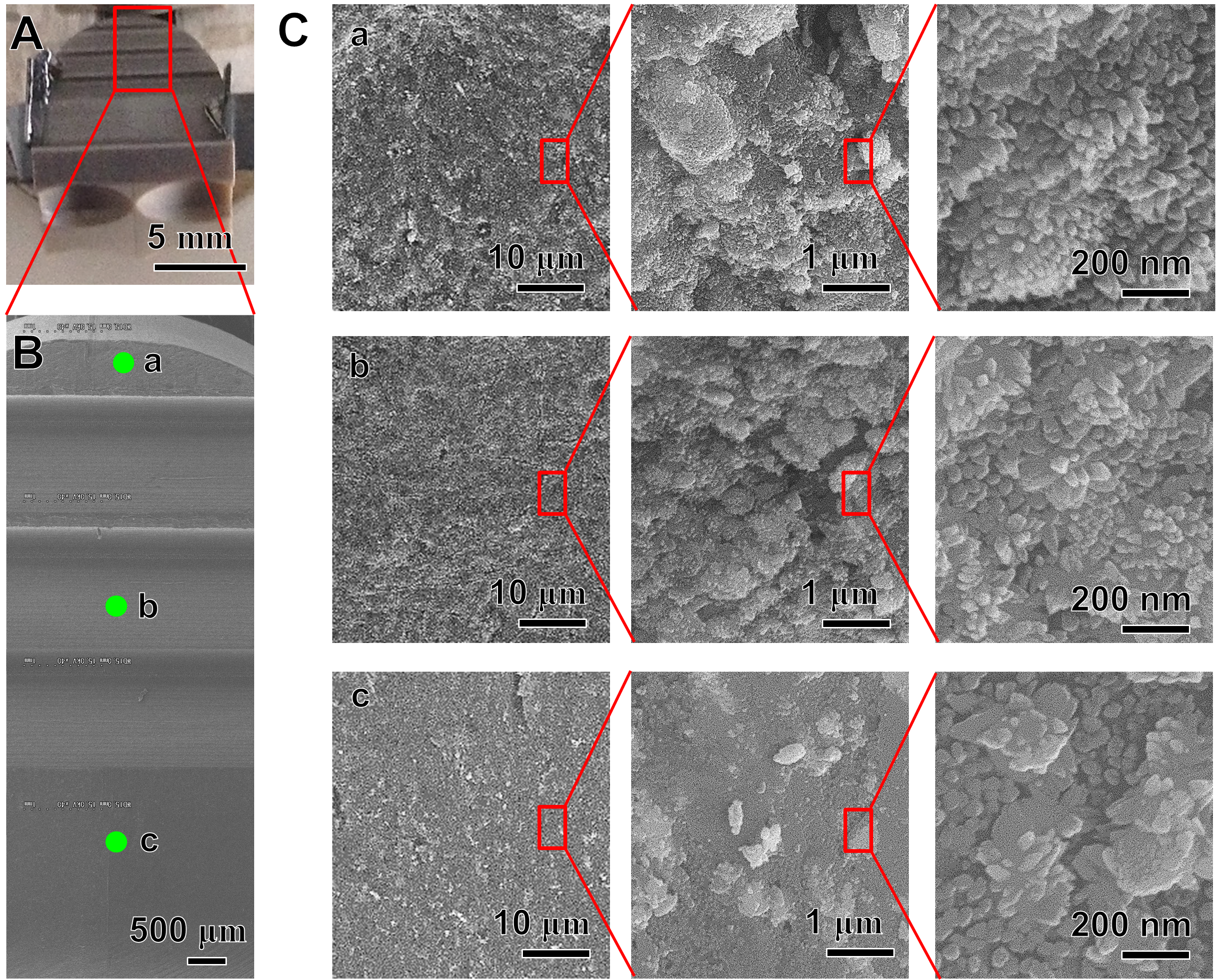

Supplement: S1 File — (A) Macroscopic view. (B) SEM image (C) Magnified SEM images on each point (a, b, and c). Nano-scale roughness was observed on each point. (TIF) [file pone.0184495.s001.tif]
